# Overexpression of Cohesion Establishment Factor DSCC1 through E2F in Colorectal Cancer

**DOI:** 10.1371/journal.pone.0085750

**Published:** 2014-01-17

**Authors:** Kiyoshi Yamaguchi, Rui Yamaguchi, Norihiko Takahashi, Tsuneo Ikenoue, Tomoaki Fujii, Masaru Shinozaki, Giichiro Tsurita, Keisuke Hata, Atsushi Niida, Seiya Imoto, Satoru Miyano, Yusuke Nakamura, Yoichi Furukawa

**Affiliations:** 1 Division of Clinical Genome Research, Advanced Clinical Research Center, Institute of Medical Science, The University of Tokyo, Tokyo, Japan; 2 Laboratory of Sequence Analysis, Human Genome Center, Institute of Medical Science, The University of Tokyo, Tokyo, Japan; 3 Department of Surgery, Research Hospital, Institute of Medical Science, The University of Tokyo, Tokyo, Japan; 4 Laboratory of DNA Information Analysis, Human Genome Center, Institute of Medical Science, The University of Tokyo, Tokyo, Japan; 5 Laboratory of Molecular Medicine, Human Genome Center, Institute of Medical Science, The University of Tokyo, Tokyo, Japan; H. Lee Moffitt Cancer Center & Research Institute, United States of America

## Abstract

Ctf18-replication factor C complex including Dscc1 (DNA replication and sister chromatid cohesion 1) is implicated in sister chromatid cohesion, DNA replication, and genome stability in *S. cerevisiae* and *C. elegans*. We previously performed gene expression profiling in primary colorectal cancer cells in order to identify novel molecular targets for the treatment of colorectal cancer. A feature of the cancer-associated transcriptional signature revealed from this effort is the elevated expression of the proto-oncogene *DSCC1*. Here, we have interrogated the molecular basis for deviant expression of human DSCC1 in colorectal cancer and its ability to promote survival of cancer cells. Quantitative PCR and immunohistochemical analyses corroborated that the expression level of DSCC1 is elevated in 60–70% of colorectal tumors compared to their matched noncancerous colonic mucosa. An *in silico* evaluation of the presumptive *DSCC1* promoter region for consensus DNA transcriptional regulatory elements revealed a potential role for the E2F family of DNA-binding proteins in controlling DSCC1 expression. RNAi-mediated reduction of E2F1 reduced expression of DSCC1 in colorectal cancer cells. Gain- and loss-of-function experiments demonstrated that DSCC1 is involved in the viability of cancer cells in response to genotoxic stimuli. We reveal that E2F-dependent expression of DSCC1 confers anti-apoptotic properties in colorectal cancer cells, and that its suppression may be a useful option for the treatment of colorectal cancer.

## Introduction

Colorectal cancer (CRC) is one of the most frequent human neoplasms in the world. In CRC cells, disruption of systems governing genetic or epigenetic integrity renders different features such as chromosomal instability (CIN), microsatellite instability (MSI), and CpG island methylator phenotype (CIMP). A great majority of colorectal tumors exhibit CIN that includes gross genetic changes such as deletions, amplifications, inversions, rearrangements, gain or loss of whole or large portions of chromosomes, and translocations [1]. An earlier study identified somatic mutations in five genes including *MRE11*, *ZW10*, *ZWILCH*, *ROD*, and *DING*, among 100 human CIN-candidate genes that shared similarity with yeast or fly “instability” genes [2]. Their data suggested that at least one of three functions including double-strand break repair, kinetochore function, and chromatid segregation, is impaired in CIN tumors by somatic mutation. Another study searched for mutations of 102 human homologues of yeast CIN genes in 132 colorectal cancers. Consequently, they identified a total of 11 mutations in five genes that included four associated with sister chromatid cohesion (*SMC1L1*, *CSPG6*, *NIPBL*, and *STAG3*, the homologues of yeast *SMC1*, *SMC3*, *SCC2*, and *SCC3*, respectively) [3]. Since sister chromatid cohesion is indispensable for cellular processes such as chromosome segregation, homologous recombinational repair, and regulation of transcription [4], genetic alterations in the components and regulators should play a crucial role in the CIN of colorectal tumors.

We previously performed gene expression profile analysis in CRC [5], and identified that DNA replication and sister chromatid cohesion 1 (*DSCC1*, also known as *DCC1*) was frequently elevated in colorectal tumors compared with non-cancerous colonic mucosa. Dcc1p, a homolog of DSCC1, was first identified as a member of alterative replication factor C (RFC) complex in the yeast, and physically associates with Ctf8p and Ctf18p [4]. Deletion of the component, Ctf18p, Ctf8p, or Dcc1p, resulted in severe sister chromatid cohesion defects, and increased sensitivity to microtubule depolymerizing drugs, suggesting that these components are essential for maintenance of chromatin integrity [4]. Although Dcc1p was not essential for the viability of yeast, deletion of Dcc1p led to synthetic lethality in combination with mutation of other sister chromatid cohesion proteins [6]. In addition to the implication in sister chromatid cohesion, the CTF18-DSCC1-CTF8-RFC complex plays a crucial role in DNA replication through the interaction with single-stranded and primed DNA as a loader of proliferating cell nuclear antigen [7]. Furthermore, genetic network analysis of functionally related genes in the yeast suggested that the components of CTF18-DSCC1-CTF8-RFC complex interact with the MAD/BUB spindle checkpoint pathway, the RAD51 DNA repair pathway for double-strand breaks, the RAD9 DNA damage checkpoint, and the TOF1/MRC DNA replication checkpoint pathway [8], [9]. The finding that mutation in CTF18-RFC increased triplet repeat instability corroborated the role of this complex in the DNA-replication checkpoint [10]. These data indicated that DSCC1 plays an important role in replication, spindle checkpoint and DNA repair, which prompted us to investigate whether deregulated expression of DSCC1 is involved in human colorectal tumorigenesis.

Here, we show for the first time that DSCC1 is frequently up-regulated in CRC at least in part through the enhanced transcriptional activation by E2F. We also reveal that elevated expression of DSCC1 confers chemoresistance in CRC cells by providing tumor cells with anti-apoptotic properties. These findings will contribute to a better understanding of CRC, and serve as a starting point for the development of novel strategies for diagnosis and treatment of CRC.

## Materials and Methods

### Ethics statement

This project was approved by the ethical committee of Institute of Medical Science, the University of Tokyo (IMSUT-IRB, 21-14-0806). Written informed consent was obtained from all patients in this study. All colorectal cancer tissues and corresponding non-cancerous tissues were obtained from surgical specimens of patients who underwent surgery.

### Cell culture

Human CRC cell lines HCT116, HCT-15, SW480, DLD-1, LoVo, Caco-2, LS174T, HT-29, and RKO were purchased from the American Type Culture Collection (Manassas, VA). All cells were grown in appropriate media supplemented with FBS (Life Technologies, Carlsbad, CA) and antibiotic/antimycotic solution (Sigma, St. Louis, MO).

### Preparation of plasmids expressing DSCC1 and E2Fs

The entire coding region of *DSCC1* cDNA (GenBank accession No. NM_024094) was amplified by RT-PCR using a set of primers; forward primer: 5′-CCGGAATTCATGAAGAGGACCCGCGAC-3′ and reverse primer: 5′-CGGCTCGAGAGAAATGGGTCTTCTCGAATTAT-3′ (underlined nucleotides indicate the recognition sites of restriction enzymes). The PCR products were cloned into the *Eco*RI and *Xho*I sites of pcDNA3.1/myc-His. We additionally generated plasmids expressing HA-tagged DSCC1 (pCAGGS-DSCC1). The constructs pcDNA3-HA-E2Fs were kindly provided by Dr. J. R. Nevins (Duke University, Durham, NC).

### Quantitative PCR and gene copy number analysis

Real-time PCR was performed using the LightCycler 480 system (Roche Diagnostics, Indianapolis, IN). Genomic DNAs were extracted from CRC cell lines for copy number analysis. Quantitative PCR was performed on ABI PRISM 7900HT Sequence Detection System, using FAM-labeled probes (5′-TCAGGTTTCCTACCTTCCGGCTGCTT-3′) and a set of primers (forward: 5′-GGCGCGCTTTCAAACG-3′, reverse: 5′-GCGGGCAAGAAAGAAGTTCC-3′) for *DSCC1*, and TaqMan Copy Number Reference Assays RNase P as a quantitative control (Life Technologies). The copy number of *DSCC1* in the cancer cells was calculated in comparison with genomic DNA from healthy volunteers using CopyCaller Software.

### Subcellular fractionation and immunoblotting

Cells were lysed in radioimmunoprecipitation assay buffer (50 mM Tris-HCl, pH 8.0, 150 mM NaCl, 0.5% sodium deoxycholate, 1% Nonidet P-40, 0.1% SDS) supplemented with a Protease Inhibitor Cocktail Set III (Calbiochem, San Diego, CA). Nuclear extracts were prepared using Nuclear Extract Kit (Active Motif, Carlsbad, CA). Proteins were separated by SDS-PAGE and immunoblot analysis was performed using the indicated antibodies. Horseradish peroxidase-conjugated goat anti-mouse or anti-rabbit IgG (GE Healthcare, Buckinghamshire, UK) served as the secondary antibody for the ECL Detection System (GE Healthcare).

### Immunostaining

Primary antibodies used for immunohistochemical and immunocytochemical staining were anti-DSCC1 (B01P, Abnova, Taipei, Taiwan) and anti-Myc (Sigma). The specificity of DSCC1 antibody was confirmed by the blocking with DSCC1 recombinant protein (data not shown). These experiments were performed as described previously [11].

### Induction of apoptosis and flow cytometry

To study the induction of apoptosis, cells were treated with camptothecin (Wako, Osaka, Japan), doxorubicin (LC Laboratories, Woburn, MA), MG132 (Merck Millipore, Darmstadt, Germany), or exposed to γ-irradiation (Gammacell 40, Atomic Energy of Canada, Ontario, Canada). Expression of cleaved poly (ADP-ribose) polymerase (PARP) and cleaved caspase-3 was detected by western blot analysis using anti-cleaved PARP (9541) and anti-caspase-3 antibodies (9662), respectively (Cell Signaling Technology, Danvers, MA). Assessment of apoptosis was also performed by annexin V and PI double-staining using Alexa Fluor 488 Annexin V/Dead Cell Apoptosis Kit (Life Technologies). Briefly, cultured cells were treated with vehicle or camptothecin for 24 h. The cells were stained with Annexin V and PI, and subsequently analyzed on a FACSCalibur (Becton Dickinson, Franklin Lakes, NJ) using FlowJo software (Tree Star, Ashland, OR).

### Cell viability assay

Plasmids expressing short hairpin RNA (shRNA) using U6 promoter (psiU6BX3.0) were prepared as described previously [12]. Plasmids expressing DSCC1 shRNA (psiU6-shDSCC1) were constructed by cloning double-stranded oligonucleotides into the *Bbs*I sites of the psiU6BX3.0 vector. Two target sequences, 5′-GUGGACAGAAGAAGAUAUU-3′ (shDSCC1#1) and 5′-GCAAACCAUAGGUGCAUUA-3′ (shDSCC1#2), were used for DSCC1 shRNAs. As negative controls, we prepared a plasmid targeting enhanced green fluorescent protein (psiU6-shEGFP) and those targeting scrambled sequences of shDSCC1#1 (5′-AAAUUGCGAAGGUGAUGAA-3′; psiU6-shDSCC1#1scr) or shDSCC1#2 (5′-AACACGUUAAUAACCGGUG-3′; psiU6-shDSCC1#2scr). Cell viability assays were carried out as described previously using HCT116, SW480, and RKO cells transfected with plasmids expressing shEGFP, shDSCC1, or scramble shDSCC1 [11]. To investigate the effect of DSCC1 overexpression on cell proliferation, we transfected SW480 and HCT116 cells with pCAGGS-DSCC1 and established two or three clones stably expressing exogenous DSCC1. Control SW480 and HCT116 cells transfected with empty vector were also established as mock cells.

### Promoter reporter assays and site-directed mutagenesis

Luciferase reporter plasmids containing the *DSCC1* promoter were prepared by cloning the 5′-flanking region of *DSCC1* into the *Mlu*I and *Bgl*II restriction enzyme sites of pGL3-Basic vector (Promega, Madison, WI). A DNA fragment of approximately 1.0-kb in the 5′-flanking region of *DSCC1* was amplified by PCR using genomic DNA from healthy volunteers and a set of primers (forward: 5′-CGACGCGTATGTCTGCTCAGATCCTTTGAAT-3′, reverse: 5′-GAAGATCTCGCCGGGTCTAGGAGTCC-3′). Mutant plasmids containing substitutions in putative E2F binding sites of the *DSCC1* promoter were generated by site-directed mutagenesis using the QuikChange II XL Site-Directed Mutagenesis Kit (Agilent Technologies, Santa Clara, CA). Cells seeded into 6-well plates were transfected with the reporter plasmids together with pRL-TK (Promega) using FuGENE 6 reagent. Cells were harvested 24 hours after transfection, and reporter activities were measured by dual luciferase system (TOYO B-Net, Tokyo, Japan). For the knockdown of E2F1 expression, synthetic E2F1 siRNA was purchased from Sigma (sense: 5′-GGGAGAAGUCACGCUAUGA-3′, antisense: 5′-AUAGCGUGACUUCUCCCCC-3′).

### Chromatin immunoprecipitation assay

To investigate the interaction of E2F1 with the *DSCC1* promoter region, a chromatin immunoprecipitation (ChIP) assay was performed according to the Agilent Mammalian ChIP protocol with slight modifications. HCT116 cells were cross-linked with 1% formaldehyde for 10 min at room temperature and quenched with 0.4 M glycine. Chromatin extracts were sheared by micrococcal nuclease digestion, and subsequently protein-DNA complexes were immunoprecipitated with 3 µg of anti-E2F1 polyclonal antibody (C-20, Santa Cruz Biotechnology, Santa Cruz, CA) bound to anti-rabbit IgG-coated Dynabeads (Life Technologies). Non-immune rabbit IgG (Santa Cruz Biotechnology) was used as a negative control. The precipitated DNAs were subjected to quantitative PCR analysis with a primer set (forward (−26) 5′-CCGGAAACACGCCCATGGC-3′ and reverse (+127) 5′-GGGTCCTCTTCATCGCAGC-3′) to amplify the *DSCC1* promoter region. Specificity of the assay was determined by the amplification of a distal upstream region in the *DSCC1* promoter with the following primers: forward (−1279) 5′-AGTTGTAGGGAATGTTTCCCATT-3′ and reverse (−1111) 5′-GATTGGTTCATGTGACCTACTTC-3′. In addition, the amplifications of cell division cycle 2 (*CDC2*) promoter and glyceraldehyde-3-phosphate dehydrogenase (*GAPDH*) promoter were used for positive and negative controls, respectively (primers: *CDC2* forward 5′-CGCCCTTTCCTCTTTCTTTC-3′, *CDC2* reverse 5′-ATCGGGTAGCCCGTAGACTT-3′, *GAPDH* forward 5′-TACTAGCGGTTTTACGGGCG-3′, *GAPDH* reverse 5′-TCGAACAGGAGGAGCAGAGAGCGA-3′).

## Results

### Expression of DSCC1 is frequently elevated in CRC

**Figure 1:**
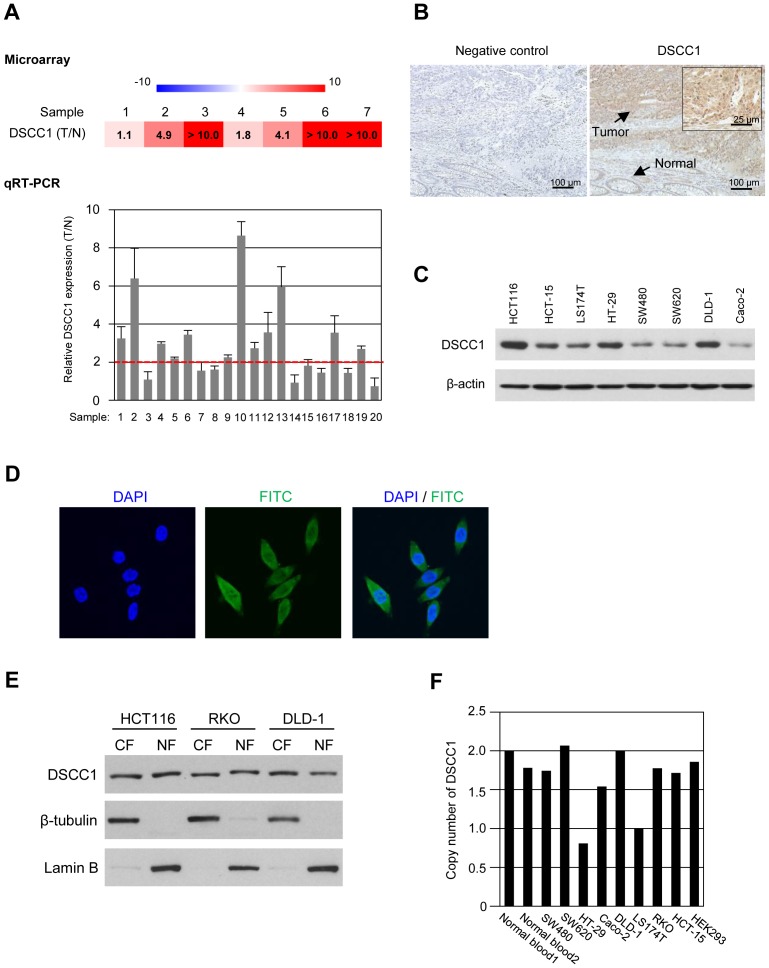
Expression of DSCC1 in colorectal tumors. (A) Relative expression ratios of *DSCC1* in seven colorectal cancer tissues to their corresponding normal tissues in our microarray data (upper panel). Relative expression levels of *DSCC1* in an additional 20 colorectal tumors and the corresponding non-cancerous mucosa was analyzed by quantitative PCR (lower panel). Quantity of *DSCC1* was normalized to *HPRT1* expression. Y axis indicates the ratio of mean of *DSCC1* expression in tumor to that in their corresponding normal tissue. The data represents mean ± SD from triplicate experiments. (B) Representative image of immunohistochemical staining of DSCC1 in a human colon cancer tissue containing cancer cells and adjacent normal mucosa. (C) Expression of DSCC1 in CRC cell lines was detected by western blotting using anti-DSCC1 antibody. (D) HCT116 cells were probed with anti-DSCC1 antibody followed by FITC-conjugated anti-mouse IgG secondary antibody (green). Nuclei were counter-stained with DAPI (blue). (E) HCT116, RKO, and DLD-1 cells were separated into the cytoplasmic (CF) and nuclear fractions (NF), and the cytoplasmic and nuclear proteins were subjected to SDS-PAGE followed by western blotting. Purity of the fractions was determined by the presence of β-tubulin (cytoplasmic marker) and lamin B (nuclear marker). (F) Copy number analysis of *DSCC1* in eight CRC cell lines and HEK293 cells. Relative copy number of *DSCC1* gene was determined by quantitative PCR using *RPPH1* as an endogenous reference. The copy number was calculated by dividing their PCR products by those of peripheral leukocytes from healthy volunteers, and subsequently multiplying by 2.

In order to identify novel target molecules for the treatment and/or diagnostic biomarkers of CRC, we previously performed expression profile analysis of colorectal tumors and their matched normal colorectal tissues by cDNA microarray [5]. Among the genes deregulated in colorectal tumors, expression of DNA replication and sister chromatid cohesion 1 (*DSCC1*) was increased more than two-fold in 5 of 7 colorectal cancers compared with corresponding non-cancerous colon mucosa (Figure 1A). Subsequent real-time PCR analysis using an additional 20 CRC tissues and the corresponding non-cancerous mucosa revealed that *DSCC1* expression was elevated more than two-fold in 12 of the 20 tumors (Figure 1A). An immunohistochemical staining showed accumulated DSCC1 protein in 29 of 40 CRC tissues compared with corresponding adjacent non-cancerous colonic mucosa (Figure 1B). Although we searched for correlations between its expression and clinicopathological factors including age and sex of the patients, location, size, and histological data of the tumors such as depth of invasion, lymph node involvement, and vascular or lymphatic vessel invasion, none of the factors was significantly associated with DSCC1 expression (Table S1). Additionally, western blot analysis using CRC cell lines revealed that DSCC1 was abundantly expressed in HCT116, HT-29, and DLD-1 cells, and that it was expressed at low levels in SW480, SW620, and Caco-2 cells (Figure 1C). Although we compared stability of DSCC1 protein in HCT116 (DSCC1-high) and SW480 (DSCC1-low) cells by cycloheximide chase assay, DSCC1 was relatively stable in both HCT116 and SW480 cells. Treatment with MG132, a proteasome inhibitor also did not enhance DSCC1 expression (Figure S1A). These data suggested that protein stability is not likely to play a major role in the elevated expression of DSCC1 in cancer cells.

Immunohistochemical analysis unexpectedly depicted accumulated DSCC1 in the cytoplasm and nucleus of DSCC1-positive cancer cells (Figure 1B), although Dscc1 was reported to play a role in the establishment of cohesion during DNA replication in the yeast. To elucidate its subcellular localization, we carried out immunocytochemical staining of endogenous DSCC1 in HCT116 cells. Consistent with the immunohistochemical staining of cancer tissues, DSCC1 protein was localized in both cytoplasm and nucleus (Figure 1D and S1B). Furthermore, western blot analysis using cytoplasmic and nuclear fractions extracted from HCT116, RKO, and DLD-1 cells (Figure 1E) and cells expressing Myc-tagged DSCC1 confirmed its subcellular localization in the cytoplasm as well as the nucleus (Figure S1C and S1D).

### Copy number analysis of *DSCC1*

To address whether gene amplification is involved in the DSCC1 overexpression, we conducted copy number analysis by quantitative PCR using RNase P as a control. Compared with peripheral leukocytes from healthy volunteers, the copy number of *DSCC1* was not increased in any CRC cell lines tested (Figure 1F). It is worth noting that a decrease in copy number was observed in HT-29 cells that abundantly expressed DSCC1 (Figure 1C). We further analyzed copy number alteration of *DSCC1* in colon and rectum adenocarcinoma (The Cancer Genome Atlas Colorectal Cancer project) using the cBioPortal database (http://www.cbioportal.org/public-portal/). As a result, putative copy number changes were found in 7 of 257 colorectal adenocarcinomas (2.7%), suggesting that amplification of *DSCC1* does not play a major role in the enhanced DSCC1 expression.

### Regulation of *DSCC1* promoter activity

**Figure 2:**
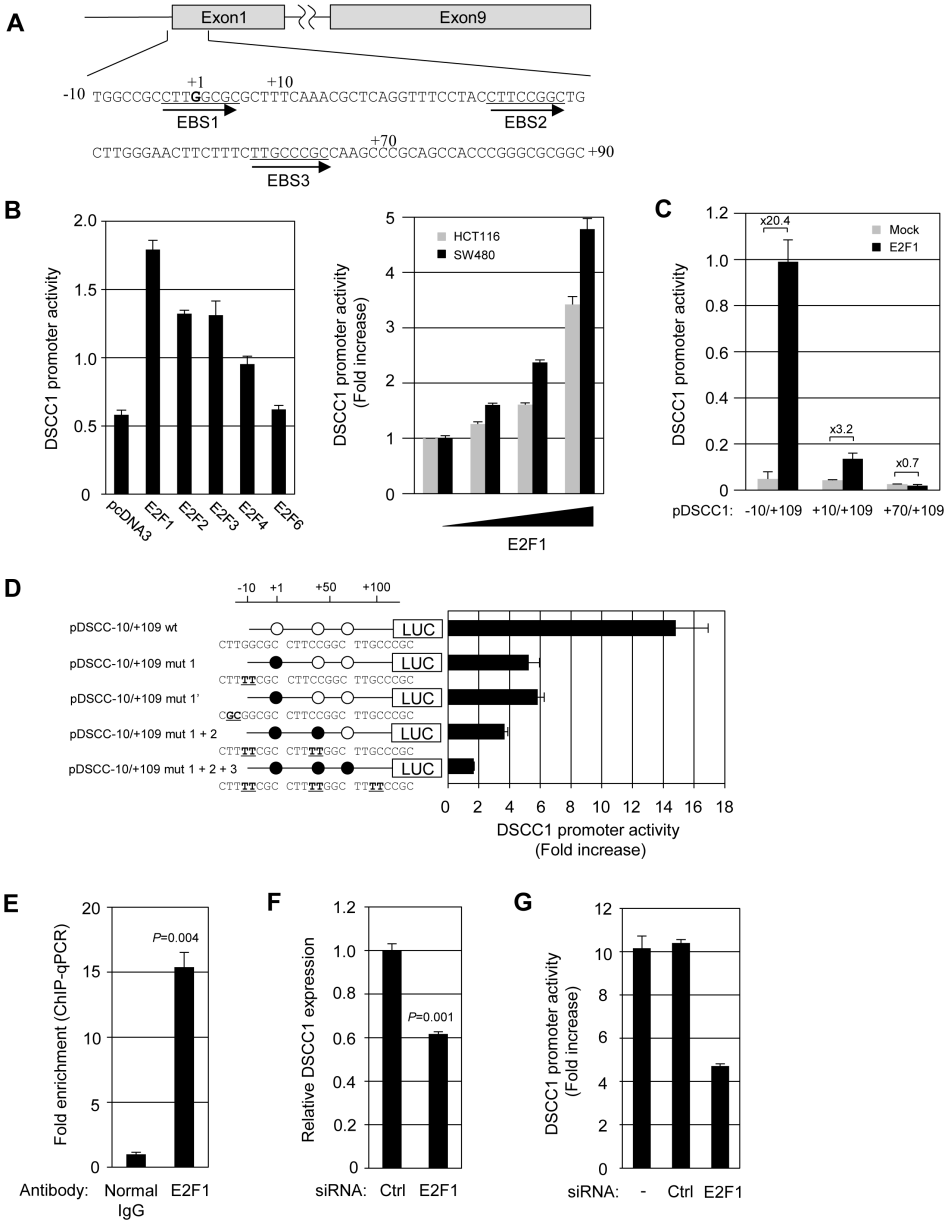
Regulation of *DSCC1* promoter activity by E2F transcription factor. (A) Nucleotide sequence of the −10 to +90 bp region human *DSCC1*. Three putative E2F binding motifs are underlined. (B) pDSCC1-1023/+109 was transiently transfected with pRL-TK and pcDNA3 HA-E2Fs into SW480, or with pRL-TK and pcDNA3 HA-E2F1 (0.01-1 µg) into SW480 and HCT116 cells. (C) pDSCC1-10/+109 or the shorter promoter constructs was transfected with E2F1 or the empty vector into SW480 cells. (D) Site-directed mutation analysis of putative E2F binding sites in the proximal promoter region. pDSCC1-10/+109 or its mutant clones was transfected with E2F1 or the empty vector into SW480 cells. The data represents mean ± SD from three independent experiments. Promoter activity indicates the relative luciferase unit or the fold induction over empty vector transfectant. (E) Chromatin immunoprecipitation was performed using anti-E2F1 antibody. The precipitated DNAs were subjected to the amplification of *DSCC1* promoter by quantitative PCR. To ascertain the specific binding to the EBS, the amplification of a distal upstream region in the *DSCC1* promoter was used for normalization. A significant difference was determined by t-test. (F) HCT116 cells were transfected with control or E2F1 siRNA (25 nM) for 48 h. *DSCC1* expression was detected by quantitative PCR. A significant difference was determined by t-test. (G) SW480 cells were transfected with control or E2F1 siRNA (25 nM), followed 8 h later by transfection with reporter plasmid (pDSCC1-10/+109) and E2F1 expression vector or the empty vector. After 48 h, luciferase activity was measured. The data represents mean ± SD from three independent experiments.

To resolve the mechanism of elevated DSCC1 expression in CRC, we investigated the promoter activity of *DSCC1* in HCT116 cells. Reporter assay using plasmids containing a 5′-flanking region of *DSCC1* (pDSCC1-1023/+109) showed that this region has a substantial promoter activity (data not shown). In the region, we identified a putative E2F-binding motif, EBS1 (−3/+5; 5′-CTTGGCGC-3′) using the JASPAR (http://jaspar.genereg.net/) and TFSEARCH databases (http://www.cbrc.jp/research/db/TFSEARCH.html) (Figure 2A). This putative binding site shared high similarity with the consensus motif for E2F, TTTSSCGC with S  =  G or C. Since E2F transcription factors are frequently deregulated in a variety of tumors, we tested the effect of E2Fs on the *DSCC1* promoter activity. Although E2F1, E2F2, E2F3, and E2F4 increased the promoter activity, E2F6 did not change the activity. E2F1, which showed the strongest induction among the four members, augmented the activity in a dose-dependent fashion (Figure 2B and S2A). This enhancement was also observed in other cell lines including LoVo, HeLa, and HEK293 (data not shown). To examine the possible involvement of EBS1 in the enhancement, we measured reporter activity using the constructs pDSCC1-10/+109 and +10/+109 in the presence or absence of E2F1 (Figure 2C). Basal reporter activities of these reporter plasmids were not significantly different in the absence of E2F1 plasmids. Deletion of EBS1 (pDSCC1+10/+109) dramatically decreased E2F1-induced reporter activity (from 20.4-fold to 3.2-fold). Unexpectedly, enhancement of reporter activity by E2F1 was still observed in +10/+109. Further deletion up to +70 of the promoter (pDSCC1+70/+109) completely diminished E2F1-induced reporter activity (Figure 2C). In agreement with this result, we found two additional presumptive EBSs, EBS2 (+31/+38; 5′-CTTCCGGC-3′) and EBS3 (+57/+64; 5′-TTGCCCGC-3′) in the region between +10 and +70. To address responsibilities of EBS1, EBS2, and EBS3 for the induction, we prepared four mutant reporter constructs (Figure 2D) by substituting the GC-rich segment in E2F consensus motifs, TTTSSCGC (S  =  C or G) or STTTS, because these core motifs were reportedly crucial for E2F binding [13], [14]. Compared with wild type pDSCC1-10/+109 (14.8-fold induction), both types of EBS1-mutant plasmids (pDSCC1-10/+109 mut1, and mut1′) remarkably decreased the reporter activity in response to E2F1 (5.2-fold and 5.8-fold, respectively). Mutations in both EBS1 and EBS2 (pDSCC1-10/+109 mut1+2) further decreased in the E2F1-induced activity (3.7-fold). Mutant reporter plasmid containing substitutions in the three elements (pDSCC1-10/+109 mut1+2+3) almost diminished the enhancement (1.7-fold), suggesting that the three E2F binding motifs are responsible for the regulation of *DSCC1* promoter activity.

### Interaction of E2F1 with the *DSCC1* promoter region

To determine whether E2F1 binds to the promoter region of *DSCC1*, we performed quantitative ChIP assay using anti-E2F1 antibody and a set of primers encompassing the three putative E2F-binding elements. The promoter of cell division cycle 2 gene (*CDC2*), a well-known E2F1 target, was enriched 13.4-fold with the immunoprecipitated DNA (Figure S2B). Expectedly, the *DSCC1* promoter region was enriched by up to 15.4-fold in the DNA, suggesting an interaction of the *DSCC1* promoter region with E2F1 (Figure 2E).

**Figure 3:**
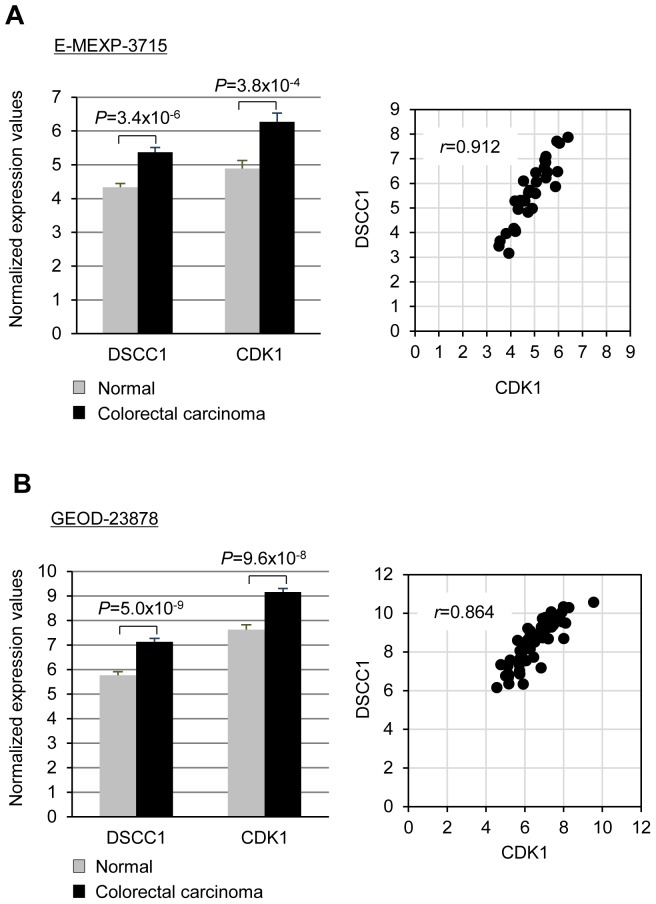
Positive correlation between *DSCC1* and *CDK1* expression in colorectal tumors. This association was shown by two sets of microarray data, E-MEXP-3715 (A) and GEOD-23878 (B), in the Gene Expression Atlas database (http://www.ebi.ac.uk/gxa/). The Pearson's correlation coefficient (r) between *DSCC1* and *CDK1* expression values was then calculated to assess their correlation.

To confirm the involvement of E2F1 in regulating DSCC1 expression, we investigated the silencing effect of E2F1 on DSCC1 expression. Real-time PCR and western blot analyses showed that depletion of E2F1 decreased DSCC1 expression (Figure 2F and S2C). RNAi-mediated knockdown of E2F1 activity was confirmed by reporter assay showing significant reduction of the *DSCC1* promoter activity from 10.4 (Ctrl siRNA) to 4.7-fold by E2F1 siRNA in SW480 cells (Figure 2G). These results suggested that *DSCC1* transactivation is, at least in part, regulated by E2F1 in CRC through its interaction with the *DSCC1* promoter region, and that the three EBSs play an important role in the transcriptional activation. To further investigate whether DSCC1 expression is modulated by E2F transcriptional activity, we compared the relative expression of *DSCC1* with *CDK1* (*CDC2*), as readout of E2F transcriptional activity, using two independent data sets (E-MEXP-3715 and GEOD-23878) in the Gene Expression Atlas database (http://www.ebi.ac.uk/gxa/). In the data sets, *DSCC1* and *CDK1* were significantly up-regulated in colorectal tumors compared with normal colonic tissues (Figure 3). Notably, both data sets calculated high values of correlation coefficient (E-MEXP-3715, *r* = 0.912 and GEOD-23878, *r* = 0.864) between *DSCC1* and *CDK1*, supporting the view that *DSCC1* is another downstream gene regulated by E2F.

### Effect of DSCC1 on the proliferation of CRC cells

**Figure 4:**
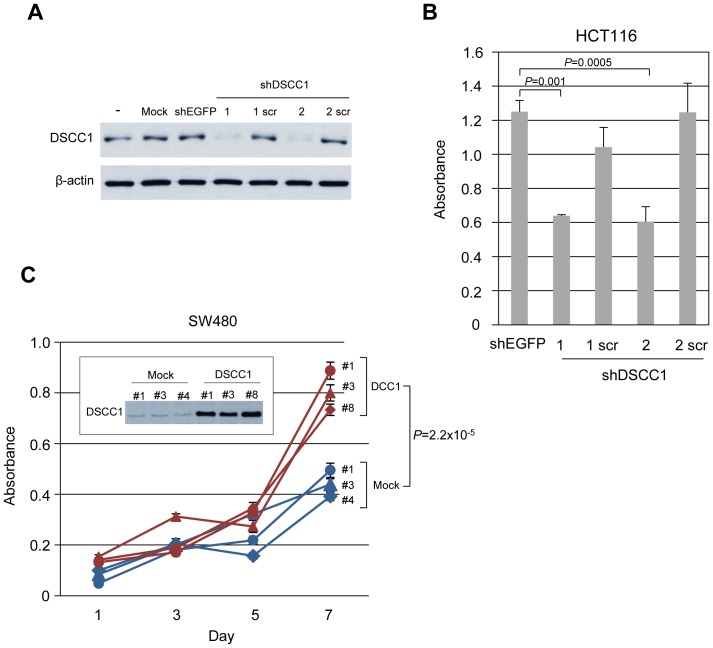
Involvement of DSCC1 in CRC cell proliferation. (A) HCT116 cells were transfected with control (Mock and EGFP) and DSCC1 shRNAs for 48 h using Nucleofector kit, and western blot analysis was performed. Expression of β-actin served as a control. (B) Viability of cells transfected with shRNAs was measured by WST-8 assay. The data represents mean ± SD from three independent transfections. P values were calculated with the Dunnett's test for multiple comparisons to shEGFP-transfected cells. (C) Overexpression of DSCC1 in SW480 cells was confirmed by western blotting using anti-DSCC1 antibody. Equivalent number of three mock and three DSCC1 cells was plated in 96-well plates, and cell proliferation assays were performed at the indicated time points. The data represents mean ± SD from five experiments. A significant difference between mock and DSCC1 cells was determined by two-way repeated measures ANOVA.

To address the role of its elevated expression in CRC cells, we investigated whether DSCC1 is involved in the proliferation of cancer cells. We carried out cell viability assay using plasmids expressing both DSCC1 shRNA (shDSCC1#1, or shDSCC1#2) and neomycin resistant gene. Plasmids containing the scrambled sequence of DSCC1 shRNAs (shDSCC1#1scr and shDSCC1#2scr) and plasmid containing EGFP shRNA (shEGFP) served as controls. Transfection with these DSCC1 shRNAs (shDSCC1#1, or shDSCC1#2) reduced the expression of DSCC1, whereas transfection with controls (shEGFP, shDSCC1#1scr and shDSCC1#2scr) had no effect (Figure 4A). HCT116 cells were cultured in media containing appropriate concentration of G418 and the cell viability was measured. We found that the number of viable cells transfected with DSCC1#1 or DSCC1#2 shRNA was significantly decreased compared to those transfected with EGFP, DSCC1#1scr, or DSCC1#2scr shRNA, indicating that DSCC1 plays a role in the viability of cancer cells (Figure 4B). Consistent data was obtained in SW480 and RKO cells (Figure S3A). These results were confirmed in repeated experiments.

In addition, we established SW480 cells that constitutively express exogenous DSCC1, and compared their proliferation with control cells transfected with mock vector (Figure 4C). Consistent with the DSCC1-knockdown data, cells expressing exogenous DSCC1 showed augmented cell proliferation compared to parental SW480 cells or control cells (*p* = 2.2×10^−5^). Similarly, exogenous DSCC1-expression enhanced the proliferation of HCT116 cells (Figure S3B).

### Role of DSCC1 in the induction of apoptosis

**Figure 5:**
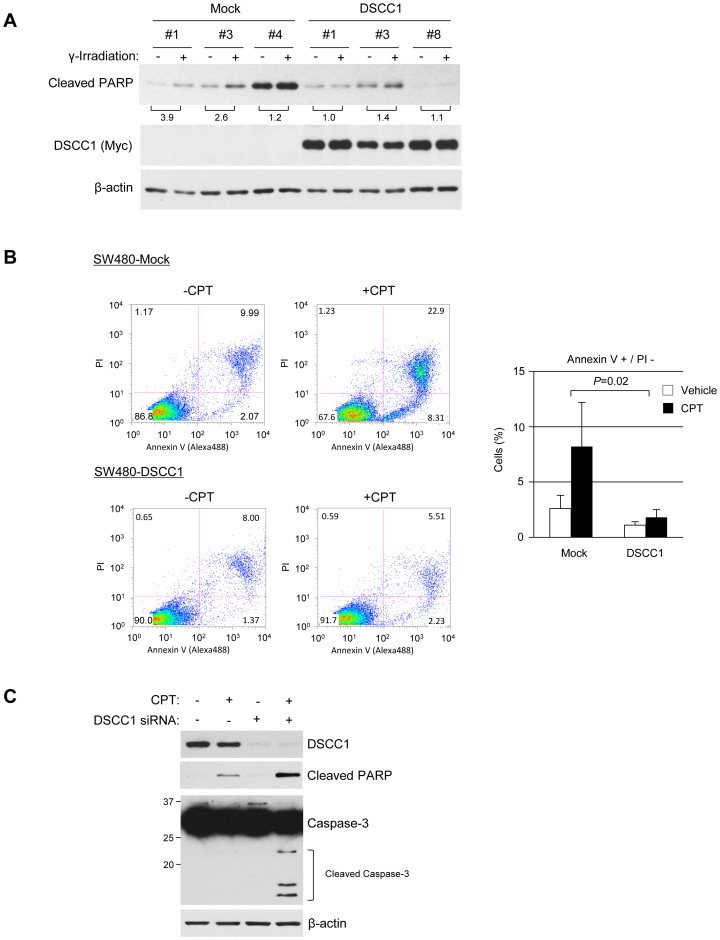
DSCC1 alters sensitivity to apoptotic stimuli. (A) SW480 cells stably expressing DSCC1 or mock (empty vector) were exposed to γ-irradiation (5 Gy). The cells were harvested 24 h after exposure, and the lysates were subjected to western blot analysis. (B) SW480 cells stably expressing DSCC1 or mock were treated with camptothecin (CPT, 30 µM). The cells were harvested 24 h after treatment, and the cell suspensions were subjected to annexin V staining. The data represents mean ± SD from three different clones. Increased annexin V-positive cell population by treatment with CPT was compared between control (Mock) and DSCC1-expressing cells. A significant difference was determined by t-test. (C) HCT116 cells were transfected with control or DSCC1 siRNA, and treated with CPT (30 µM) at 48 h. The cells were harvested 24 h after the CPT-treatment, and the lysates were subjected to western blot analysis.

Since E2F1 conferred resistance to genotoxic insults [15], [16], [17], we further investigated whether elevated DSCC1 expression plays a role in the sensitivity of cancer cells to genotoxic stimuli. SW480 cells-expressing exogenous DSCC1 (SW480-DSCC1#1, #3, and #8) were exposed to γ-irradiation, and induction of apoptosis was analyzed by immunoblotting with anti-cleaved PARP antibody. As shown in Figure 5A, quantification of cleaved PARP-specific bands revealed that γ-irradiation led to 3.9, 2.6, and 1.2-fold increase of cleaved PARP in control cells (SW480-Mock#1, #3, and #4, respectively). On the other hand, 1.0, 1.4, and 1.2-fold increase was observed in response to γ-irradiation in SW480-DSCC1#1, #3, and #8 cells, respectively, suggesting that DSCC1 suppressed apoptosis by γ-irradiation. In addition, treatment with camptothecin, an inhibitor of topoisomerase I, induced early apoptotic cells (annexin V-positive and PI-low) by 5.6±2.9% in control cells (Figure 5B), while the treatment increased early apoptotic cells by only 0.7±0.3% in SW480-DSCC1 cells, indicating a significant suppression of apoptosis (*p* = 0.02). These data suggested that elevated DSCC1 expression might confer resistance to apoptotic stimuli in cancer cells. In complete agreement with these results, knockdown of DSCC1 potentiated camptothecin- and γ-irradiation-induced apoptosis in HCT116 cells (Figure 5C and S3C). To address whether DSCC1 expression affects cell death induced by other types of cytotoxic reagents, we treated HCT116 cells with doxorubicin, a DNA intercalator, or MG132, a proteasome inhibitor, and measured the cleavage of PARP and caspase-3, indicators of apoptosis. Suppression of DSCC1 augmented the cleavage of PARP and caspase-3 in response to doxorubicin, whereas knockdown of DSCC1 did not increase the cleavage in response to MG132 (Figure S3D and S3E). Therefore, DSCC1 may be associated with the cell death caused by DNA-damage, but not with the death by other types of cytotoxic insults. Of note, DSCC1 depletion also enhanced induction of apoptosis by γ-irradiation in p53-null HCT116 cells (Figure S3F), suggesting that DSCC1-mediated resistance against apoptosis was independent of p53. These results imply that suppression of DSCC1 may be useful for the treatment and/or chemosensitization of CRC cells.

## Discussion

Regulated by the retinoblastoma tumor suppressor, pRB, and/or related proteins, E2Fs play crucial roles in cell cycle, nucleotide synthesis, DNA replication, DNA repair, and apoptosis [18], [19]. Activities of E2Fs are regulated by integration of signals transduced from the cellular DNA and the external environment. E2F1 is thought to act as an oncogene and a tumor suppressor, with its action dependent on the cellular context. Indeed, overexpression of E2F1 is observed in CRC cells, suggesting its tumorigenic role in the cancer [20]. E2F1 expression is elevated in lung metastasis of colon cancer, and correlates with thymidylate synthase expression, resulting in chemoresistance [21]. On the other hand, E2F1 is over-expressed in colon tumors with increased apoptosis and low proliferation [22]. Therefore, clarification of E2F1 targets should give a clue how this versatile transcription factor family is involved in colorectal carcinogenesis. We have demonstrated here that DSCC1 is frequently up-regulated in CRCs through the transactivation by the E2F family of transcription factors. Although *DSCC1* is located at chromosomal region 8q, which is one of the most frequently amplified chromosomal regions in colorectal tumors [23], copy number gain or amplification was not involved in DSCC1 up-regulation. Instead, we showed here that the flanking region of *DSCC1* transcription start site containing three E2F regulatory sites (EBS1, 2, and 3) plays a role in the transcriptional activation. A previous genome-wide ChIP-chip analysis reported that 20,000–30,000 E2F1-biding sites are distributed over the human genome, of which 51% overlapped transcription start sites [24]. The localization of EBS1, 2, and 3 is compatible with their findings. Among the three sites, EBS1 and EBS3 contain identical sequence with the core E2F1-binding motif (C/GC/GCGC), but EBS2 contains a 1-bp mismatch to the motif. Comparison of human and mouse *DSCC1* 5′-flanking sequences determined that EBS1, located at the −3 to +5 bp region, was well-conserved between the two species (Figure S4A). Consistent with these data, the mutation in EBS1 most remarkably reduced the E2F1-induced promoter activity among the three. Regarding other regulatory elements, we identified a region between −40 and −20 that was associated with basal promoter activity of *DSCC1* (Figure S4B). A search of transcription factor-binding elements in this region found a GC box encompassing a putative ELK1-binding site, but our reporter assay did not show significant change of the promoter activity by ELK1 (data not shown), suggesting that ELK1 may not be involved in the regulation of DSCC1.

Eight members of the mammalian E2F family have been recognized and characterized. Among these members, E2F1, E2F2, and E2F3 are categorized as transcriptional activators, and E2F4, E2F5, E2F6, E2F7, and E2F8 are categorized as repressors [25], [26]. In our promoter assay, E2F1, E2F2, E2F3, and E2F4 induced *DSCC1* promoter activity, but E2F6 did not, showing an inconsistent result with E2F4 in the transcription. Molecular studies have uncovered that E2Fs target genes are regulated not only by their binding to DNA element(s) but also by their interacting proteins such as Rb, p107, p130, polycomb group proteins, and histone-modification enzymes. Therefore, other factor(s) might affect the elevated promoter activity by E2F4. Although the direct association of E2Fs and their cofactors with the three binding sites needs future detailed analysis, the region containing the three should play a vital role in the elevated expression of DSCC1.

We here showed for the first time that DSCC1 plays an important role in survival of human cancer cells, since enhanced expression of DSCC1 induced survival of cancer cells in response to γ-irradiation, topoisomerase I inhibitor, and DNA-intercalator. The data are consistent with the finding that Dscc1 mutants exhibit sensitivity to γ-irradiation in *Saccharomyces cerevisiae* [27], [28]. Another study showed that repair of a topoisomerase I inhibitor-induced DNA double-strand breaks, required components of chromatid cohesion including *Csm3*, *Tof1*, *Mrc1*, and *Dscc1* [29]. Alternatively, DSCC1 may enhance the recombination repair through the CTF18-RFC complex. Our study additionally showed that this resistance seems to be independent of p53 because the induction of apoptosis was also potentiated in HCT116 p53−/− cells (Figure S3F). Associated with CTF8, DSCC1 forms an alternate RFC with CTF18, and further stabilizes 7-subunit complex with RFC2, RFC3, RFC4, and RFC5. Depletion of DSCC1 reduces expression of CTF18, induces decreased replication fork, increases collapse, and suppresses recovery of forks to replication inhibitors, suggesting that DSCC1 is important for DNA replication and recovery from genotoxic insults [30].

Global gene-gene interaction studies have helped gain insights into the complex genetic networks in the yeast. These studies disclosed synthetic lethal combinations of genetic dysfunction, where two genetic variations that have individually no effect on cell viability cause cell death if combined. The concept of synthetic lethality is of great importance in creating therapeutic approaches to selectively kill cancer cells, because genetic and/or epigenetic alterations are expected in cancer cells but not in noncancerous cells. For example, PARP inhibitors have been shown to induce synthetic lethality to cancer cells with BRCA1 or BRCA2 mutations [31], [32]. Of note, McLellan and colleagues validated genetic interactions of synthetic lethality in the yeast between *ctf8*, *ctf18*, *dscc1*, *ctf4*, and *rad27* with genes required for the maintenance of chromosomal stability [6]. They additionally showed that these genetic interactions are conserved in *Caenorhabditis elegans*, suggesting the potential utility of these genes for the treatment of colorectal tumors where CIN is frequently involved in the carcinogenesis. They also showed mutations in *ctf4*, *ctf8*, *ctf18*, and *dscc1* are synthetically lethal when combined with mutations in CIN genes including *mre11*, *smc1*, *smc3*, *scc2*, and *pds1* [6]. To test whether CTF18-RFC complex may be associated with chemosensitivity, CTF18, a member of CTF18-RFC complex, was knocked down in HCT116 cells. Interestingly, silencing of CTF18 resulted in the increased cell death in response to camptothecin (Figure S4C). Although further studies on molecular mechanism(s) underlying DSCC1- as well as CTF18-mediated chemoresistance are needed, these data may imply that DSCC1 may facilitate DNA repair through homologous recombination by the regulation of this complex. If this is the case, inhibition of DSCC1 in combination with treatment inducing genotoxic insults such as camptothecin and γ-irradiation may be an effective therapeutic option. Comprehension of DNA damage, repair activities, and anti-apoptotic abilities should be needed to clarify the threshold for apoptosis in each cell.

In summary, our data may give a clue to the understanding of new molecular mechanisms underlying resistance of cancer cells against genotoxic insults, and may contribute to the development of new strategies to overcome the chemoresistance to anti-cancer drugs.

## Supporting Information

Figure S1 **Subcellular localization of DSCC1.** (A) HCT116 and SW480 cells were treated with MG132 (10 µM, 6 h) or cycloheximide (100 µg/ml). The cells were harvested at the indicated time points, and the lysates were subjected to western blot analysis. (B) High magnification images of Figure 1D (x180). (C) HCT116 cells expressing Myc-tagged DSCC1 were probed with anti-Myc antibody followed by FITC-conjugated anti-mouse IgG secondary antibody (green). Nuclei were counter-stained with DAPI (blue). (D) The cytoplasmic and nuclear proteins were analyzed by western blotting.
(TIF)

Figure S2 **Regulation of DSCC1 by E2Fs.** (A) HEK293T cells were transfected with pcDNA3 HA-E2F1, -E2F2, -E2F3, -E2F4, and -E2F6 for 24 h, and the lysates were subjected to western blot analysis. (B) Chromatin immunoprecipitation was performed using anti-E2F1 antibody. The precipitated DNAs were subjected to the amplification of *CDC2* promoter by quantitative PCR. (C) HeLa cells were transfected with control or E2F1 siRNA (25 nM) for 48 h. Western blot analysis was performed using the indicated antibodies.
(TIF)

Figure S3 **DSCC1 alters response to genotoxic insults.** (A) Viability of cells transfected with shRNAs was measured by WST-8 assay. The data represents mean ± SD from three to five independent transfections. P values were calculated with the Dunnett's test for multiple comparisons to shEGFP-transfected cells. (B) Overexpression of DSCC1 in HCT116 cells was confirmed by western blot analysis with anti-Flag antibody. Equivalent number of two mock clones, two DSCC1 clones, and parental HCT116 cells was plated in 96-well plates, and these cells were cultured in medium containing 0.5% FBS. Cell proliferation assays were performed at the indicated time points. The data represents mean ± SD from eight experiments. (C) HCT116 cells were treated with control or DSCC1 siRNA (10 nM), followed 48 h later by exposure to γ-irradiation (5 Gy). (D, E) HCT116 cells were treated with control or DSCC1 siRNA (10 nM), followed 48 h later by treatment with doxorubicin (5 µM) or MG132 (2 µM). (F) HCT116 p53-/- cells were treated with control or DSCC1 siRNA (10 nM), followed 48 h later by exposure to γ-irradiation (5 Gy). The cells were harvested 24 h after exposure, and the lysates were subjected to western blot analysis.
(TIF)

Figure S4 **Alignment of human and mouse** ***DSCC1*** **5′-flanking sequence.** (A) Alignment of human and mouse *DSCC1* 5′-flanking sequence by the DBTSS database (http://dbtss.hgc.jp/). Top strand represents the 5′-flanking sequences of human *DSCC1*, and the bottom strand represents the 5′-flanking sequences of mouse *Dscc1*. E2F binding motifs are underlined. (B) pDSCC1-133/+109 or the shorter promoter constructs was transfected with pRL-TK into SW480 cells. The promoter activity was measured by luciferase activity. Each value represents mean ± SD from three independent transfections. (C) The effect of CTF18 siRNA (S: 5′-CCAACUGCCUGGUCAUCG-3′, AS: 5′-UCGAUGACCAGGCAGUUG-3′) was evaluated by quantitative PCR (CTF18 primers, forward: 5′-CTTCTCGGTGTGGCAGGA-3′, reverse: 5′-CAGCAGGAGTGTGTCAGCAG-3′). HCT116 cells were treated with control or CTF18 siRNA (10 nM), followed 48 h later by treatment with CPT (30 µM). The cells were harvested 24 h after treatment, and the lysates were subjected to western blot analysis.
(TIF)

Table S1 **Correlations between DSCC1 expression and the clinicopathological characteristics of the 40 colon cancer patients.**
(XLS)
