# Supplementary figures and images for: Overexpression of Cohesion Establishment Factor DSCC1 through E2F in Colorectal Cancer
Source: PLoS One. 2014 Jan 17;9(1):e85750. doi: 10.1371/journal.pone.0085750 (PMC3894995; doi:10.1371/journal.pone.0085750)

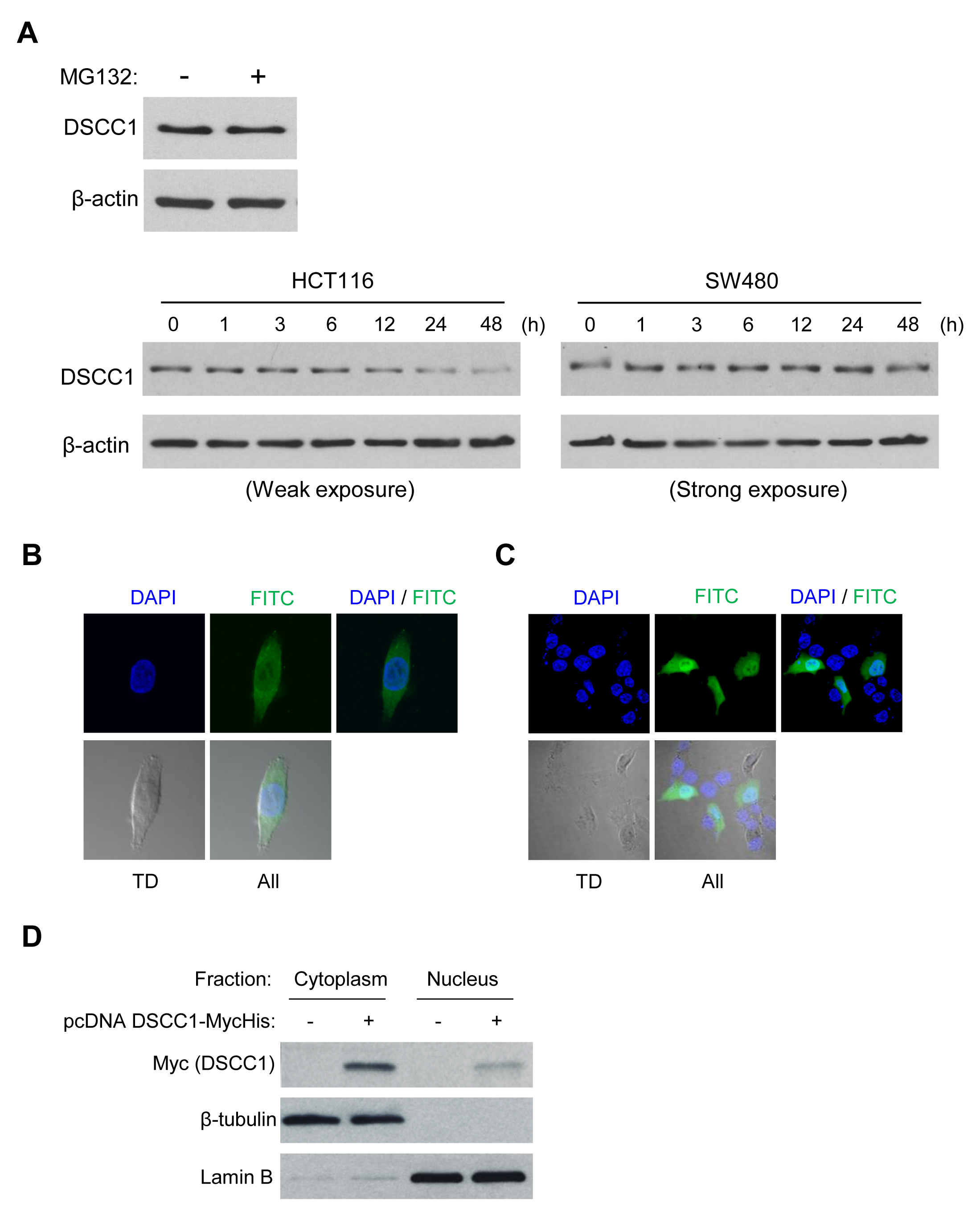

Supplement: Figure S1 — Subcellular localization of DSCC1. (A) HCT116 and SW480 cells were treated with MG132 (10 µM, 6 h) or cycloheximide (100 µg/ml). The cells were harvested at the indicated time points, and the lysates were subjected to western blot analysis. (B) High magnification images of Figure 1D (x180). (C) HCT116 cells expressing Myc-tagged DSCC1 were probed with anti-Myc antibody followed by FITC-conjugated anti-mouse IgG secondary antibody (green). Nuclei were counter-stained with DAPI (blue). (D) The cytoplasmic and nuclear proteins were analyzed by western blotting. (TIF) [file pone.0085750.s001.tif]

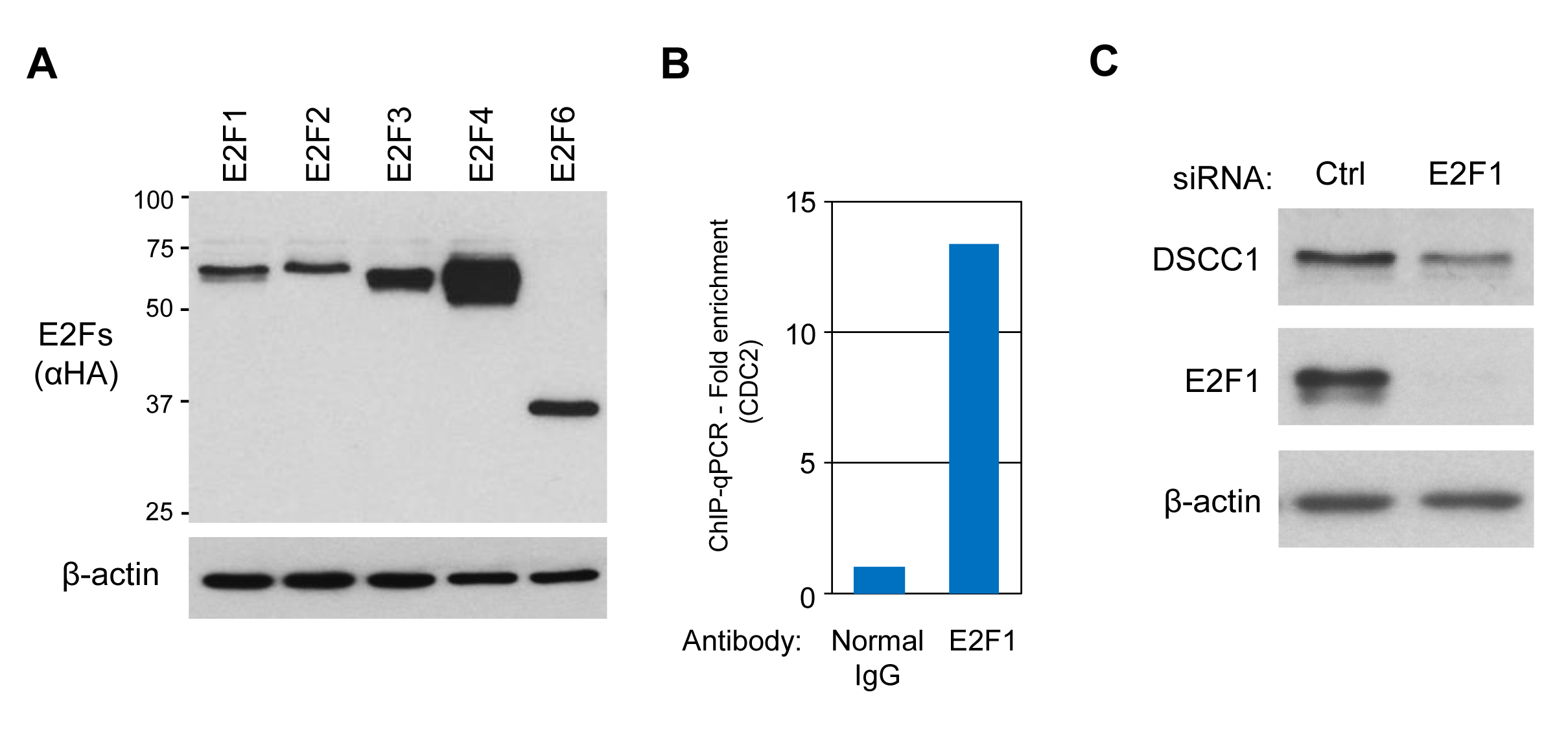

Supplement: Figure S2 — Regulation of DSCC1 by E2Fs. (A) HEK293T cells were transfected with pcDNA3 HA-E2F1, -E2F2, -E2F3, -E2F4, and -E2F6 for 24 h, and the lysates were subjected to western blot analysis. (B) Chromatin immunoprecipitation was performed using anti-E2F1 antibody. The precipitated DNAs were subjected to the amplification of CDC2 promoter by quantitative PCR. (C) HeLa cells were transfected with control or E2F1 siRNA (25 nM) for 48 h. Western blot analysis was performed using the indicated antibodies. (TIF) [file pone.0085750.s002.tif]

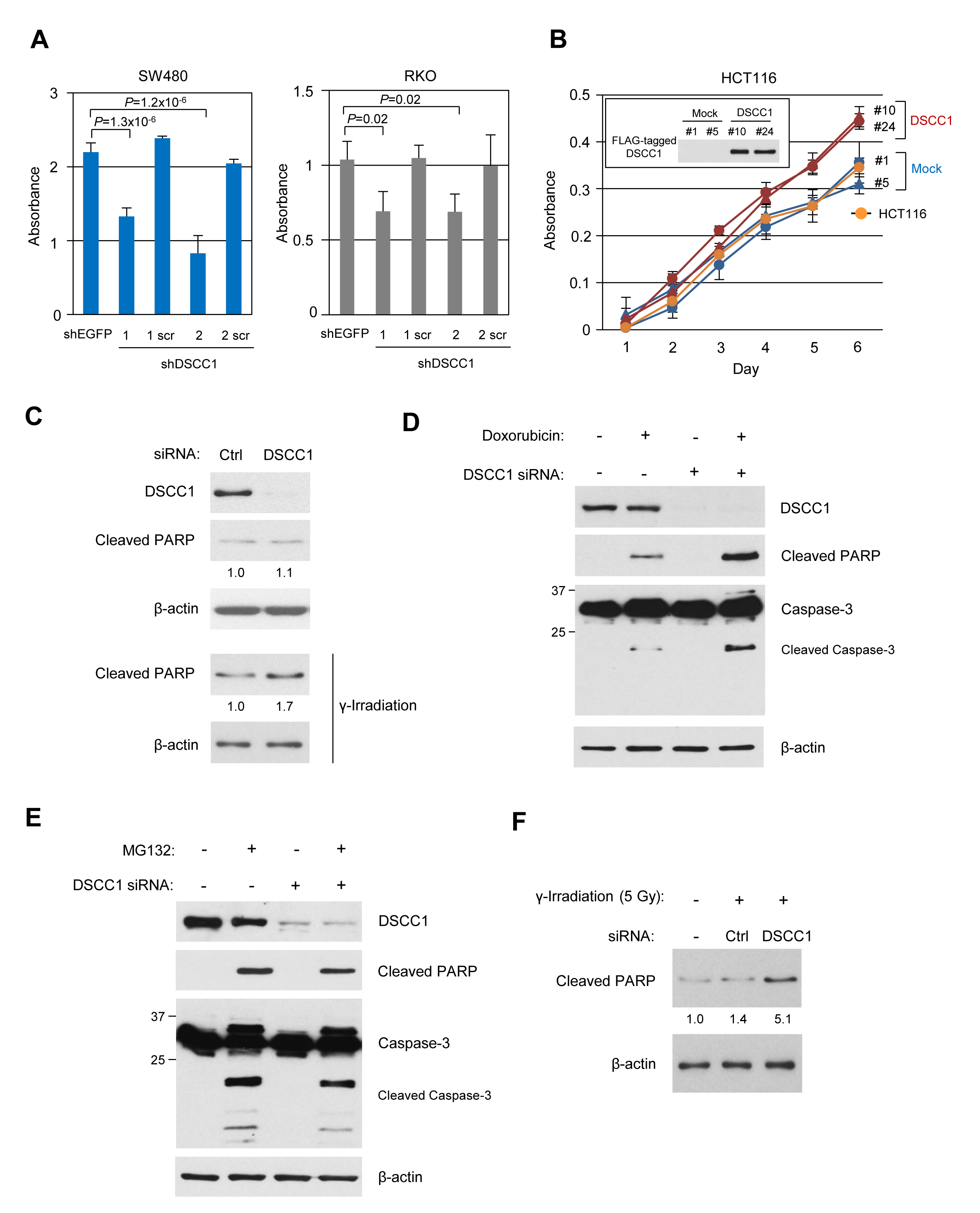

Supplement: Figure S3 — DSCC1 alters response to genotoxic insults. (A) Viability of cells transfected with shRNAs was measured by WST-8 assay. The data represents mean ± SD from three to five independent transfections. P values were calculated with the Dunnett's test for multiple comparisons to shEGFP-transfected cells. (B) Overexpression of DSCC1 in HCT116 cells was confirmed by western blot analysis with anti-Flag antibody. Equivalent number of two mock clones, two DSCC1 clones, and parental HCT116 cells was plated in 96-well plates, and these cells were cultured in medium containing 0.5% FBS. Cell proliferation assays were performed at the indicated time points. The data represents mean ± SD from eight experiments. (C) HCT116 cells were treated with control or DSCC1 siRNA (10 nM), followed 48 h later by exposure to γ-irradiation (5 Gy). (D, E) HCT116 cells were treated with control or DSCC1 siRNA (10 nM), followed 48 h later by treatment with doxorubicin (5 µM) or MG132 (2 µM). (F) HCT116 p53-/- cells were treated with control or DSCC1 siRNA (10 nM), followed 48 h later by exposure to γ-irradiation (5 Gy). The cells were harvested 24 h after exposure, and the lysates were subjected to western blot analysis. (TIF) [file pone.0085750.s003.tif]

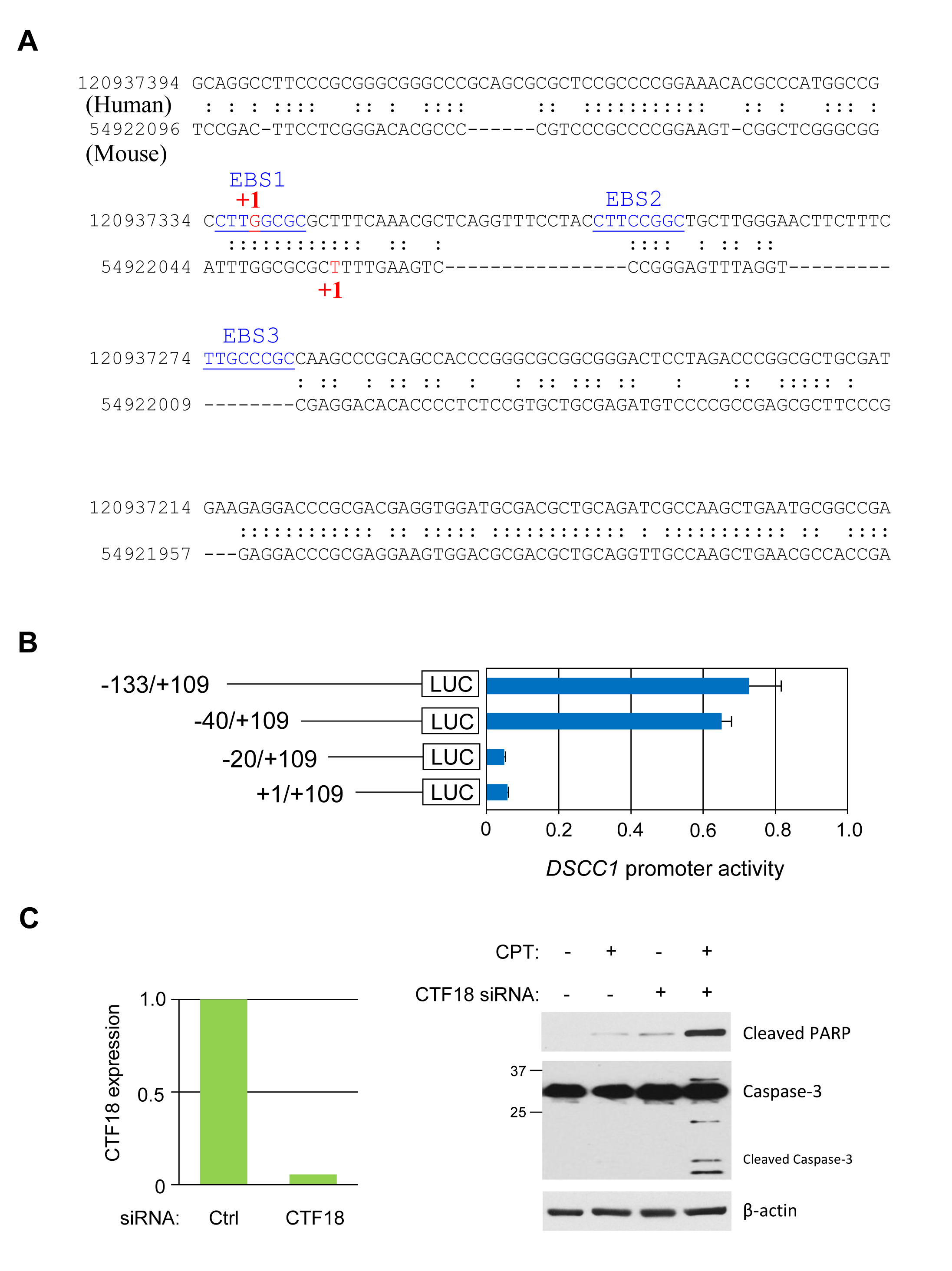

Supplement: Figure S4 — Alignment of human and mouse DSCC1 5′-flanking sequence. (A) Alignment of human and mouse DSCC1 5′-flanking sequence by the DBTSS database (http://dbtss.hgc.jp/). Top strand represents the 5′-flanking sequences of human DSCC1, and the bottom strand represents the 5′-flanking sequences of mouse Dscc1. E2F binding motifs are underlined. (B) pDSCC1-133/+109 or the shorter promoter constructs was transfected with pRL-TK into SW480 cells. The promoter activity was measured by luciferase activity. Each value represents mean ± SD from three independent transfections. (C) The effect of CTF18 siRNA (S: 5′-CCAACUGCCUGGUCAUCG-3′, AS: 5′-UCGAUGACCAGGCAGUUG-3′) was evaluated by quantitative PCR (CTF18 primers, forward: 5′-CTTCTCGGTGTGGCAGGA-3′, reverse: 5′-CAGCAGGAGTGTGTCAGCAG-3′). HCT116 cells were treated with control or CTF18 siRNA (10 nM), followed 48 h later by treatment with CPT (30 µM). The cells were harvested 24 h after treatment, and the lysates were subjected to western blot analysis. (TIF) [file pone.0085750.s004.tif]
